# OTUB1 regulation of ferroptosis and the protective role of ferrostatin-1 in lupus nephritis

**DOI:** 10.1038/s41419-024-07185-5

**Published:** 2024-11-05

**Authors:** Chen Liu, Yu-hui Gan, Wei-jing Yong, Hong-de Xu, Yong-chun Li, Hui-miao Hu, Zhan-zheng Zhao, Yuan-yuan Qi

**Affiliations:** 1 Department of Nephrology, The First Affiliated Hospital of Zhengzhou University, Zhengzhou, Henan 450052 P. R. China; 2 Zhengzhou University, Zhengzhou, Henan 450001 P. R. China; 3 Laboratory of Nephrology, The First Affiliated Hospital of Zhengzhou University, Zhengzhou, Henan 450052 P. R. China; 4 School of Pharmaceutical Sciences, Zhengzhou University, 100 Ke xue Avenue, Zhengzhou, Henan 450001 China; 5 Ministry of Education of China, Institute of Drug Discovery and Development, Zhengzhou University, 100 Kexue Avenue, Zhengzhou, Henan 450001 China

**Keywords:** Lupus nephritis, Nephritis

## Abstract

Lupus nephritis (LN) is a prevalent and severe manifestation of systemic lupus erythematosus (SLE), leading to significant morbidity and mortality. OTUB1, a deubiquitinating enzyme, has emerged as a potential therapeutic target due to its role in cellular protection and regulation of ferroptosis, a form of cell death linked to LN. Our study revealed significantly reduced OTUB1 expression in the glomeruli of LN patients and podocytes, correlated with disease severity. CRISPR/Cas9-mediated *OTUB1* knockout in podocytes resulted in pronounced injury, indicated by decreased levels of nephrin and podocin. Ferrostatin-1 treatment effectively mitigated this injury, restoring SLC7A11 expression and significantly reducing MDA levels, Fe^2+^ levels, BODIPY C11 expression, and normalized cysteine and glutathione expression. In the MRL/lpr mouse model, Ferrostatin-1 significantly improved renal function decreased proteinuria, and ameliorated renal histopathological changes, including reduced glomerular endothelial swelling, mesangial cell proliferation, and leukocyte infiltration. These results underscore the protective role of Ferrostatin-1 in modulating the pathogenesis of LN. OTUB1 plays a crucial protective role against podocyte injury in LN by regulating ferroptosis. Ferrostatin-1 effectively mitigates podocyte damage induced by OTUB1 deficiency, suggesting that targeting ferroptosis could be a promising therapeutic strategy for LN.

## Introduction

Lupus nephritis (LN) is a common and severe complication of systemic lupus erythematosus (SLE) and a major cause of morbidity and mortality, predominantly affecting women and individuals of Asian, African, and Hispanic ancestries [1, 2]. The pathogenesis of LN is complex, involving the initiation of disease by immune complexes, the activation of immune responses within the kidney, and the reaction of renal parenchymal cells to this damage [3]. While we have gained some insights into the pathogenesis of LN, the specific molecular mechanisms remain elusive.

Podocyte dysfunction is a common feature of autoimmune renal diseases, including lupus nephritis, contributing to renal injury [4]. Podocytes are essential components of the glomerular filtration barrier. Damage and loss of podocytes are closely associated with the development of proteinuria, which is a significant indicator of disease progression and poor prognosis in lupus nephritis [5]. Therefore, protecting podocytes and reducing their damage may help improve clinical outcomes in LN patients. In-depth study of the protective mechanisms of podocytes not only contributes to a more comprehensive understanding of the pathogenesis of LN but also helps reveal potential therapeutic targets.

OTUB1, a deubiquitinating enzyme, plays a crucial role in the pathogenesis of several diseases through diverse mechanisms [6, 7]. It regulates protein ubiquitination, influencing cellular signal transduction, cell cycle progression, and stress response pathways [8, 9]. Aberrant OTUB1 function is implicated in various diseases, including cancer [10], neurodegenerative disorders [11], and autoimmune conditions [9]. Investigations into OTUB1 and its associated mechanisms are crucial for elucidating the molecular basis of these diseases and developing new therapeutic strategies.

In the Korean population, the SNP rs77459372 in OTUB1 is associated with uric acid levels, underscoring OTUB1’s role in kidney disease-related traits [12]. Furthermore, OTUB1 has been identified as a top-ranked deubiquitylating enzyme likely to regulate plasma membrane transport processes in renal cortical collecting duct principal cells, emphasizing its significant role in kidney physiology [13]. Although the potential role of OTUB1 in kidney diseases has been increasingly recognized, its specific role in LN remains underexplored.

In this study, we investigated the potential molecular mechanisms of OTUB1 in podocyte injury in lupus nephritis. We found that OTUB1 expression was reduced in LN podocytes. Notably, the downregulated OTUB1 was found to promote podocyte injury in LN by regulating ferroptosis. Furthermore, we examined the protective effects of the ferroptosis inhibitor Ferrostatin-1 on OTUB1-knockout podocytes and in an LN animal model. These findings provide a theoretical basis for developing new therapeutic interventions targeting ferroptosis in LN.

## Methods

### Patients and controls

In this study, we conducted immunofluorescence experiments to assess renal pathology in lupus nephritis. Kidney biopsy samples were obtained from 12 patients, categorized into two groups based on the severity of their condition: 6 patients diagnosed with Class III LN and 6 with Class IV LN, according to the International Society of Nephrology/Renal Pathology Society (ISN/RPS) 2003 classification criteria. For comparative control, normal kidney tissue samples adjacent to renal cell carcinoma were collected from an additional 6 patients who underwent nephrectomy for cancer treatment. Prior to sample collection, all participants provided written informed consent. The study protocol was designed to adhere to ethical guidelines and was approved by the Medical Ethics Committee of the First Affiliated Hospital of Zhengzhou University (approval number: 2019-KY-134). The consent process and study design complied with the principles of the Declaration of Helsinki.

### Animal models and treatment

We utilized two distinct strains of female mice to establish our animal models. A cohort of 23 female MRL/MpJ-Fas/J (MRL/lpr) mice, known for their genetic predisposition to SLE-like symptoms, was specifically procured from the Jackson Laboratory (Bar Harbor, Maine, USA). Additionally, six female C57BL/6 mice, serving as a control strain, were sourced from Beijing Vital River Laboratory Animal Technology Co., Ltd. (Beijing, China). Upon arrival, the mice were acclimated and housed under standardized conditions with a 12-hour light/dark cycle, constant temperature, and ad libitum access to a nutritionally balanced diet and water. The well-being of the animals was closely monitored, and all experimental procedures were conducted in strict accordance with the guidelines approved by the Ethics Committee of the Animal Experimental Center of Zhengzhou University (ZZU-LAC20210416).

At the age of 16 weeks, the MRL/lpr mice were initiated on a treatment regimen involving intraperitoneal injections of Ferrostatin-1 at a dosage of 1 mg/kg/day [14]. The control group received an equivalent volume of sterile saline solution to account for the procedural intervention. This treatment protocol was maintained for a duration of 4 weeks. Throughout this period, 24-hour urine samples were systematically collected using metabolic cages to assess renal function and proteinuria.

Upon reaching 20 weeks of age, the female MRL/lpr mice and their C57BL/6 counterparts were subjected to an overnight fast to standardize metabolic conditions prior to the collection of serum and kidneys. Subsequently, the kidneys were harvested for histopathological examination, which aimed to evaluate the renal structural integrity and the extent of pathological changes in the MRL/lpr mice treated with Ferrostatin-1.

To accurately assess the renal pathological changes in the mouse models, which were analogous to those observed in human LN, we conducted a comprehensive histopathological examination of the harvested kidneys. The renal tissues were processed and sectioned for subsequent staining with Hematoxylin and eosin (HE), Masson’s trichrome, Periodic acid Schiff (PAS), and PASM (PAS with Methyl Green). All histopathological evaluations were conducted by a team of experienced renal pathologists who were blinded to the experimental groups, ensuring unbiased and standardized assessments.

### Generation of CRISPR/Cas9 cell lines

To generate CRISPR/Cas9-mediated cell lines with targeted gene knockouts, we initiated our study by designing and constructing single guide RNAs (sgRNAs) through Vigene Biosciences Inc (Shandong, China). The sgRNAs were specifically designed to target the genes of interest: *OTUB1* and *MAP1LC3B*. The nucleotide sequence for the sgRNA targeting OTUB1 was 5′-CAGGTGGAGTACATGGACCG-3′, while the sequence for the sgRNA targeting MAP1LC3B was 5′-AGATCCCTGCACCATGCCGT-3′. We obtained the human podocyte cell line (HPC) from Prof. Moin Saleem, which is a well-characterized model for studying renal diseases. The HPC cells were seeded at a density of 1 × 10^6^ cells per well and cultured for 7 days to reach confluency. Subsequently, the cells were transfected with either CRISPR/Cas9-mediated sgRNA for *OTUB1* knockout (KO), CRISPR/Cas9-mediated sgRNA for *MAP1LC3B* KO, or a control vector using standard transfection protocols. Following transfection, the cells were allowed to recover for 72 h. To enrich successfully transfected cells, the culture medium was supplemented with 2 µg/ml of the selectable marker puromycin for 48 h. This step facilitated the selection of cells that had integrated the CRISPR/Cas9 construct. Surviving cells were then expanded, and genomic DNA was extracted for further analysis. To confirm the efficiency of the gene knockout, PCR primers flanking the gRNA target sites were designed and used for amplification of the targeted genomic regions. The PCR products were subjected to gel electrophoresis and sequencing to verify the presence of the desired mutations. The sequencing data were analyzed to assess the extent of gene editing and to identify successfully edited clones. Upon identification of mixed clones with successful gene knockout, single clones were isolated and further validated through PCR, gel electrophoresis, and sequencing to ensure clonal purity and homozygous knockout status.

### Isolation of immunoglobulin G from serum samples

To isolate immunoglobulin G (IgG) from serum samples, we employed a standard Protein G affinity chromatography technique. Serum samples were collected from LN patients following informed consent and ethical approval. The detailed methodology for Protein G affinity chromatography and the BCA protein assay has been previously described in our laboratory’s published work [15], where the protocols were validated for reproducibility and accuracy. The purified IgG samples were aliquoted, stored at −80 °C, and subsequently used for functional studies.

### Construction and transfection of overexpression plasmid

The coding sequence of *OTUB1* was amplified from a cDNA library derived from healthy human podocytes and subsequently cloned into the pCMV3-C-EGFP expression vector (HG16169-ACG, Sino Biological Inc). The CMV promoter within the vector drives robust expression of the inserted gene, and the EGFP (enhanced green fluorescent protein) tag allows for visualization and assessment of transfection efficiency. The integrity and orientation of the insert were confirmed by restriction enzyme digestion and DNA sequencing, ensuring the correct integration of the *OTUB1* gene into the plasmid. The constructed plasmid, named pCMV3-OTUB1-EGFP, was then prepared for transfection using an endotoxin-free plasmid purification kit to eliminate any potential contaminants that could affect cell viability.

For the transfection process, we selected the *OTUB1*-knockout human podocyte (OTUB1-KO-HPC) cell line, which was previously established in our laboratory. The cells were seeded at an optimal density to achieve 70–80% confluency on the day of transfection. The pCMV3-OTUB1-EGFP plasmid was transiently transfected into these cells using Lipofectamine 3000 reagent (Thermo Fisher Scientific, Waltham, Massachusetts, USA), following the manufacturer’s protocol for maximum transfection efficiency. Post-transfection, the cells were incubated in a CO_2_ incubator at 37 °C with 5% CO_2_ for 48 h to allow for protein expression.

The cells were then analyzed for EGFP fluorescence using a fluorescence microscope to assess transfection efficiency and the expression of the OTUB1-EGFP fusion protein. Successfully transfected cells were harvested and subjected to Western blotting to confirm OTUB1 overexpression.

### Determination of malondialdehyde, cysteine, glutathione, Fe^2+^, and BODIPY C11

Human podocytes were harvested for the quantification of malondialdehyde (MDA), cysteine, reduced glutathione (GSH), Fe^2+^, and BODIPY C11. All measurements were performed using specific assay kits, following the manufacturer’s protocols to ensure accuracy and reproducibility.

For the determination of MDA, we used the MDA assay kit (Cat# BC0025, Solarbio). Cells were mixed with the reagents provided in the kit and incubated according to the protocol. The absorbance was then measured at the specified wavelength using a microplate reader to determine the MDA concentration.

Cysteine levels were measured using the cysteine assay kit (Cat# BC0185, Solarbio). The absorbance was measured at the recommended wavelength, and cysteine concentration was calculated based on the standard curve provided in the kit manual.

Glutathione was quantified using the reduced glutathione assay kit (Cat# A006-2-1, Nanjing Jiancheng Bioengineering Institute). The resulting absorbance was measured to determine the GSH concentration.

The cellular iron content (Fe^2+^) within the podocytes was quantified using the Cell Iron Assay Kit (A039-2-1, Nanjing Institute of Bioengineering). The cells were collected under sterile conditions and processed according to the manufacturer’s guidelines to ensure precise measurement.

For the assessment of lipid peroxidation, podocytes were stained with C11-BODIPY (Catalog No. D3861, Invitrogen, USA) after washing with PBS. The cells were incubated with the C11-BODIPY staining solution according to the manufacturer’s instructions and subsequently analyzed using a CytoFLEX flow cytometer (Beckman Coulter, Brea, CA, USA).

### Western blot

The proteins were initially separated by sodium dodecyl sulfate-polyacrylamide gel electrophoresis and then transferred onto Nitrocellulose (NC) membranes. To prevent non-specific binding, the membranes were blocked with 5% non-fat dry milk for 2 h at room temperature. Following blocking, the membranes were incubated overnight at 4 °C with primary antibodies. The next day, the membranes were incubated for 2 h at room temperature with horseradish peroxidase-conjugated secondary antibodies. Protein expression was detected using an enhanced chemiluminescence detection reagent (P10100, New Cell & Molecular Biotech Co., Ltd.). The bands were visualized using an automatic exposure system, and their intensities were quantified using ImageJ software (Supplemental Material).

The primary antibodies used in this study included: rabbit anti-OTUB1 (ab270959, Abcam), rabbit anti-SLC7A11 (12691S, Cell Signaling Technology), rabbit anti-NPHS2 (ab50339, Abcam), rabbit anti-nephrin (ab58968, Abcam), and rabbit anti-α-tubulin (2144S, Cell Signaling Technology). The secondary antibody was horseradish peroxidase-labeled goat anti-rabbit IgG (IH-0011, Beijing Qiyan Biotechnology Co., Ltd.).

### Immunofluorescence staining

Immunofluorescence staining was performed to assess the expression of OTUB1, synaptopodin, and C3, as well as to detect 4-HNE levels in renal tissues. Tissue sections were first fixed and permeabilized using standard protocols. After blocking non-specific binding with 3% bovine serum albumin (GC305010, Sevir), the sections were incubated overnight at 4 °C with the following primary antibodies: anti-OTUB1 (ab270959, Abcam), anti-synaptopodin (sc-515842, SANTA CRUZ BIOTECHNOLOGY), anti-C3 (ab11862, Abcam), and 4-HNE (ab48506, Abcam). Subsequently, sections were washed and incubated with appropriate fluorescent-labeled secondary antibodies for 50 min at room temperature. To visualize nuclei, the samples were counterstained with DAPI (G1012, Sevir) before mounting with an anti-fade mounting medium. The stained slides were examined under a fluorescence microscope (Nikon CI-L, Nikon), and images were captured for analysis.

### Immunohistochemical staining

Immunohistochemical staining was conducted on renal tissues from humans, MRL/lpr mice, and C57 BL/6 mice. Antigen retrieval was performed using a citrate-based solution (G1201, Sevir). To quench endogenous peroxidase activity, the tissues were treated with 3% hydrogen peroxide (H_2_O_2_) for 20 min. Non-specific binding was blocked by incubating the tissues with 3% bovine serum albumin (GC305010, Sevir) for 30 min.

Primary antibodies were applied, and the tissues were incubated overnight at 4 °C. The next day, the tissues were incubated with secondary antibodies for 50 min. Immunoreactivity was visualized using a diaminobenzidine substrate (G1212, Sevir). The stained sections were examined and digitally scanned under a microscope.

The primary antibodies used in this study were anti-α-smooth muscle actin (ab5694, Abcam), rabbit anti-fibronectin (ab2413, Abcam), and rabbit anti-p65 (8242S, Cell Signaling Technology). The secondary antibodies were horseradish peroxidase (HRP)-conjugated goat anti-mouse IgG (GB23301, Sevir) and HRP-conjugated goat anti-rabbit IgG (GB23303, Sevir).

### TUNEL assay

The terminal deoxynucleotidyl transferase dUTP nick end labeling (TUNEL) assay was performed on samples fixed with 4% paraformaldehyde and processed into paraffin sections. The TUNEL assay (12156792910, Roche) was conducted following the manufacturer’s protocol. Images were captured using a Nikon CI-L microscope (Nikon).

### Statistical analysis

For statistical analysis, data were processed using SPSS software, version 21.0. All numerical values are presented as the mean ± standard deviation (SD). Comparisons between the two groups were made using a two-tailed Student’s *t* test, and correlations between parameters were assessed using Pearson’s correlation analysis. A *p* value of <0.05 was considered statistically significant.

## Results

### Decreased OTUB1 expression in podocytes of lupus nephritis

**Fig. 1:**
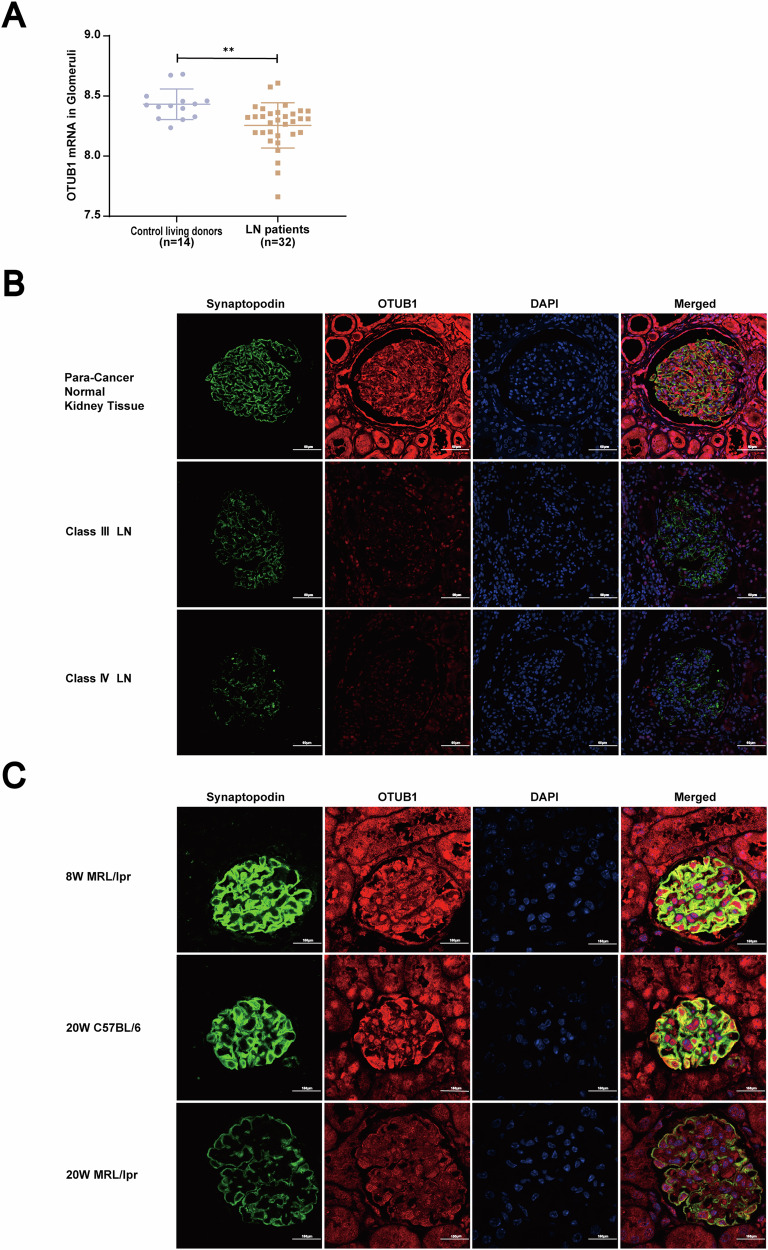
Decreased OTUB1 expression in podocytes of lupus nephritis. **A** Initial analysis of OTUB1 expression in the glomeruli of LN kidney biopsy patients was performed using the GSE32591 database. Data are represented as mean ± SD. *p < 0.05, **p < 0.01,***p < 0.001. **B** Immunofluorescence staining demonstrating co-localization of OTUB1 with the podocyte marker synaptopodin in LN patients. Scale bar: 60 μm. **C** Evaluation of OTUB1 expression in podocytes of different mouse models using immunofluorescence. Scale bar: 20 μm.

To investigate the role of OTUB1 in LN, we initially analyzed its expression in the glomeruli of LN kidney biopsy patients. Utilizing the GSE32591 database, which precisely distinguishes between glomerular and tubulointerstitial expression characteristics, we observed that OTUB1 expression in the glomeruli of LN patients was significantly lower compared to that in the control group (Fig. 1A). This initial finding prompted us to further explore the expression profile of OTUB1 in LN.

To gain a more detailed understanding, we performed a co-localization analysis of OTUB1 and synaptopodin, a podocyte-specific protein, using immunofluorescence in kidney biopsies from LN patients. The results revealed a marked decrease in the co-localization of OTUB1 with synaptopodin in the glomeruli of LN patients. This observation was accompanied by a reduction in the expression levels of both OTUB1 and synaptopodin (Fig. 1B), indicating that the decrease in OTUB1 expression might be associated with podocyte injury and loss of synaptopodin, a critical component of podocyte function and structure.

To corroborate our findings from human samples, we extended our investigation to animal models of lupus nephritis. Specifically, we examined OTUB1 expression in the renal tissues of MRL/lpr mice, a well-established murine model of lupus. Using immunofluorescence, we found that 20-week-old MRL/lpr mice, which exhibit significant renal pathology, had significantly lower OTUB1 expression in podocytes compared to both 8-week-old MRL/lpr mice without renal injury and age-matched healthy C57BL/6 mice (Fig. 1C). This reduction in OTUB1 expression in the older, disease-affected mice indicates a correlation between disease progression and OTUB1 downregulation.

Decreased OTUB1 expression is consistently observed in the glomeruli of LN patients and lupus-prone mice. This reduction, especially in podocytes, suggests a role in the pathogenesis of lupus nephritis through podocyte injury and dysfunction. Further studies are needed to elucidate the underlying mechanisms.

### Downregulation of OTUB1 expression relieved inhibition of ferroptosis promoting podocyte injury in lupus nephritis

**Fig. 2:**
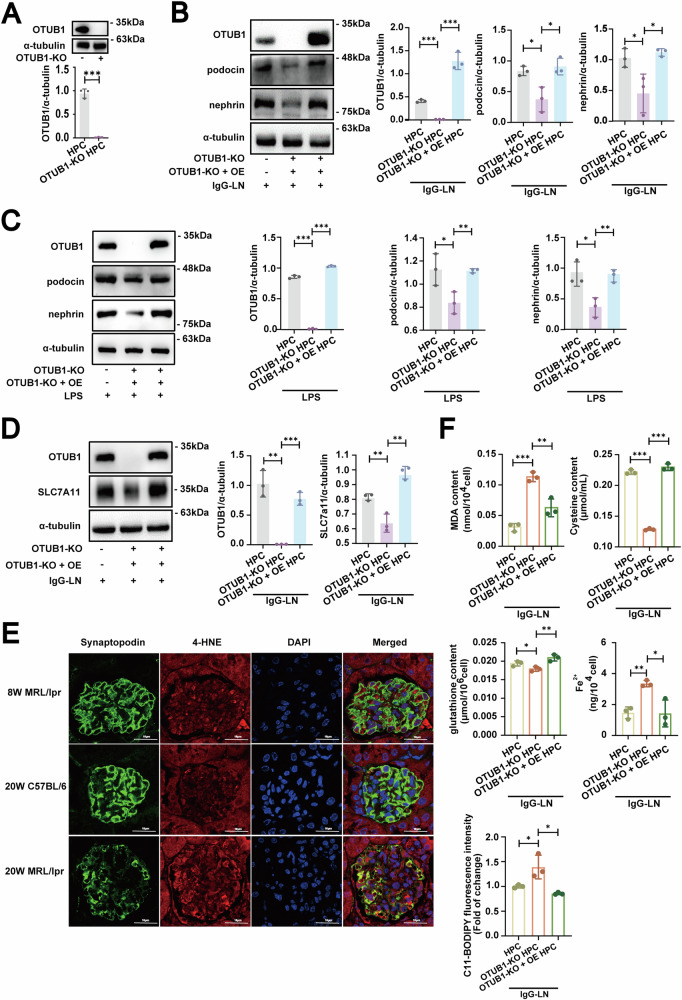
Impact of OTUB1 downregulation on podocyte injury and ferroptosis in lupus nephritis. **A** Validation of OTUB1 knockout efficiency in podocytes using CRISPR-Cas9. **B**, **C** Evaluation of podocyte marker expression after exposing OTUB1-deficient podocytes to IgG from lupus nephritis patients or LPS, mimicking autoimmune and inflammatory conditions. **D** Analysis of SLC7A11 expression in OTUB1-deficient podocytes, showing a significant drop, with re-expression of OTUB1 restoring SLC7A11 levels. **E** Co-localization of 4-HNE with synaptopodin in mouse models using immunofluorescence. Scale bar: 16 μm. **F** Assessment of ferroptosis in OTUB1 knockout podocytes, demonstrated by increased MDA levels, reduced cysteine and glutathione levels, and elevated Fe^2+^ levels and BODIPY C11 expression. Data are represented as mean ± SD. *p < 0.05, **p < 0.01,***p < 0.001.

To clarify the relationship between low expression of OTUB1 and podocyte injury, we employed *CRISPR-Cas9* technology to generate OTUB1-knockout podocytes. The efficiency of OTUB1 knockout was depicted in Fig. 2A. Due to the key pathogenic mechanisms of LN in SLE patients, which involve the deposition of immune complexes and subsequent local inflammatory responses, we extracted IgG antibodies from LN patients. These autoantibodies simulated immune-mediated damage to podocytes under pathological conditions [15, 16]. Concurrently, we employed LPS to mimic local inflammatory reactions in the kidneys. This approach enabled us to reconstruct the immunological and inflammatory mechanisms leading to podocyte injury in LN patients within an in vitro model.

In experiments using IgG derived from patients with LN or stimulated with LPS, we found that following OTUB1 deletion, there was a significant reduction in the expression of specific podocyte markers, indicating podocyte damage (Fig. 2B, C). This damage was evidenced by decreased levels of markers such as nephrin and podocin, which are critical for maintaining podocyte function and integrity. Upon re-expression of OTUB1 in these cells, restoring its levels, there was a recovery in the expression of podocyte markers, suggesting alleviation of podocyte injury (Fig. 2B, C).

Further investigation into the molecular changes in the OTUB1-knockout podocyte cell line revealed a significant decrease in the expression of SLC7A11 (Fig. 2D), a critical component of ferroptosis. SLC7A11 is essential for the import of cystine, which is crucial for the synthesis of glutathione, an important cellular antioxidant. Re-expression of OTUB1 restored SLC7A11 levels (Fig. 2D), indicating that OTUB1 regulates SLC7A11 expression.

We subsequently assessed the levels of 4-HNE, a lipid peroxidation byproduct that served as a key marker of ferroptosis. Compared to the 8-week-old MRL/lpr mice with no significant renal injury and the age-matched 20-week-old C57BL/6 mice, the 20-week-old MRL/lpr mice exhibited marked renal damage. Immunofluorescence staining revealed a significant increase in 4-HNE expression within the podocytes of the 20-week-old MRL/lpr mice (Fig. 2E), indicating an elevation in lipid peroxidation, a core process in the ferroptosis pathway.

Furthermore, OTUB1-knockout cells exhibited marked oxidative stress, as evidenced by increased MDA levels and decreased cysteine and glutathione concentrations (Fig. 2F). Elevated Fe^2+^ levels and enhanced BODIPY C11 fluorescence were also observed, indicating augmented iron accumulation and lipid peroxidation. Upon re-expression of OTUB1, MDA levels were significantly reduced, and cysteine and glutathione concentrations were restored to levels comparable to the control group. Additionally, Fe^2+^ levels and BODIPY C11 fluorescence returned to values similar to those of the control group (Fig. 2F). These results suggested that OTUB1 was critical for maintaining cellular redox homeostasis by regulating SLC7A11 expression, thereby playing a pivotal role in the modulation of ferroptosis.

### Ferrostatin-1 mitigated podocyte injury in lupus nephritis

Given the substantial role of ferroptosis in OTUB1-mediated podocyte injury in LN, we examined whether the ferroptosis inhibitor Ferrostatin-1 could ameliorate podocyte injury induced by OTUB1 downregulation.

**Fig. 3:**
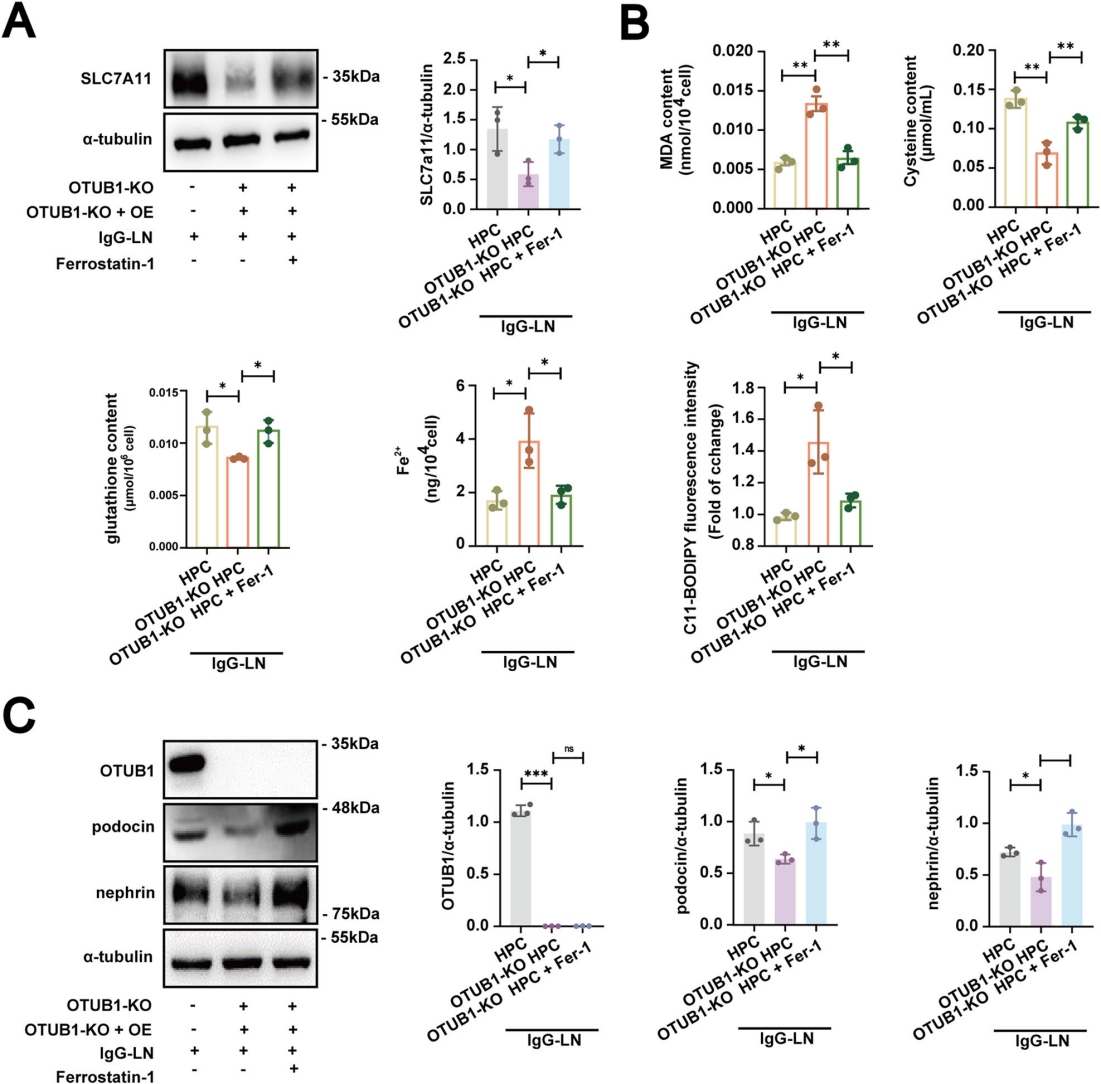
Effects of ferrostatin-1 on podocyte injury induced by OTUB1 downregulation in lupus nephritis. **A**, **B** Treatment with Ferrostatin-1 improved conditions in OTUB1-deficient podocytes, evidenced by the restoration of SLC7A11 expression, reduction in MDA levels, and increase in cysteine and glutathione levels, and elevated Fe^2+^ levels and BODIPY C11 expression. **C** Restoration of nephrin and podocin expression in podocytes treated with Ferrostatin-1. Data are represented as mean ± SD. *p < 0.05, **p < 0.01,***p < 0.001.

We first treated OTUB1 knockout podocytes with Ferrostatin-1 to assess its protective effects. Following the application of Ferrostatin-1, we observed a significant improvement in the podocytes. Notably, Ferrostatin-1 restored the expression levels of SLC7A11, which had been markedly decreased due to OTUB1 knockout (Fig. 3A). Restoring SLC7A11 expression is vital for enhancing cellular antioxidant defenses by facilitating cysteine uptake, essential for glutathione synthesis.

In addition to restoring SLC7A11 levels, treatment with Ferrostatin-1 significantly reduced MDA levels (Fig. 3B), indicating that Ferrostatin-1 effectively mitigates oxidative damage within the podocytes. Furthermore, Ferrostatin-1 treatment increased cysteine and glutathione levels (Fig. 3B), supporting the enhancement of the antioxidative capacity of the cells and their resilience to ferroptosis. Additionally, the application of this ferroptosis inhibitor resulted in a decrease in Fe^2+^ levels and BODIPY C11 expression, reinforcing the role of OTUB1 deficiency in promoting ferroptosis (Fig. 3B).

Furthermore, the expression of podocyte markers nephrin and podocin was significantly diminished in OTUB1 knockout cells. Ferrostatin-1 treatment restored the expression of these markers (Fig. 3C), indicating an alleviation of podocyte injury and potential recovery from the structural damage caused by the loss of OTUB1.

### Inhibitors of ferroptosis alleviated renal damage in MRL/lpr mice with renal injury

**Fig. 4:**
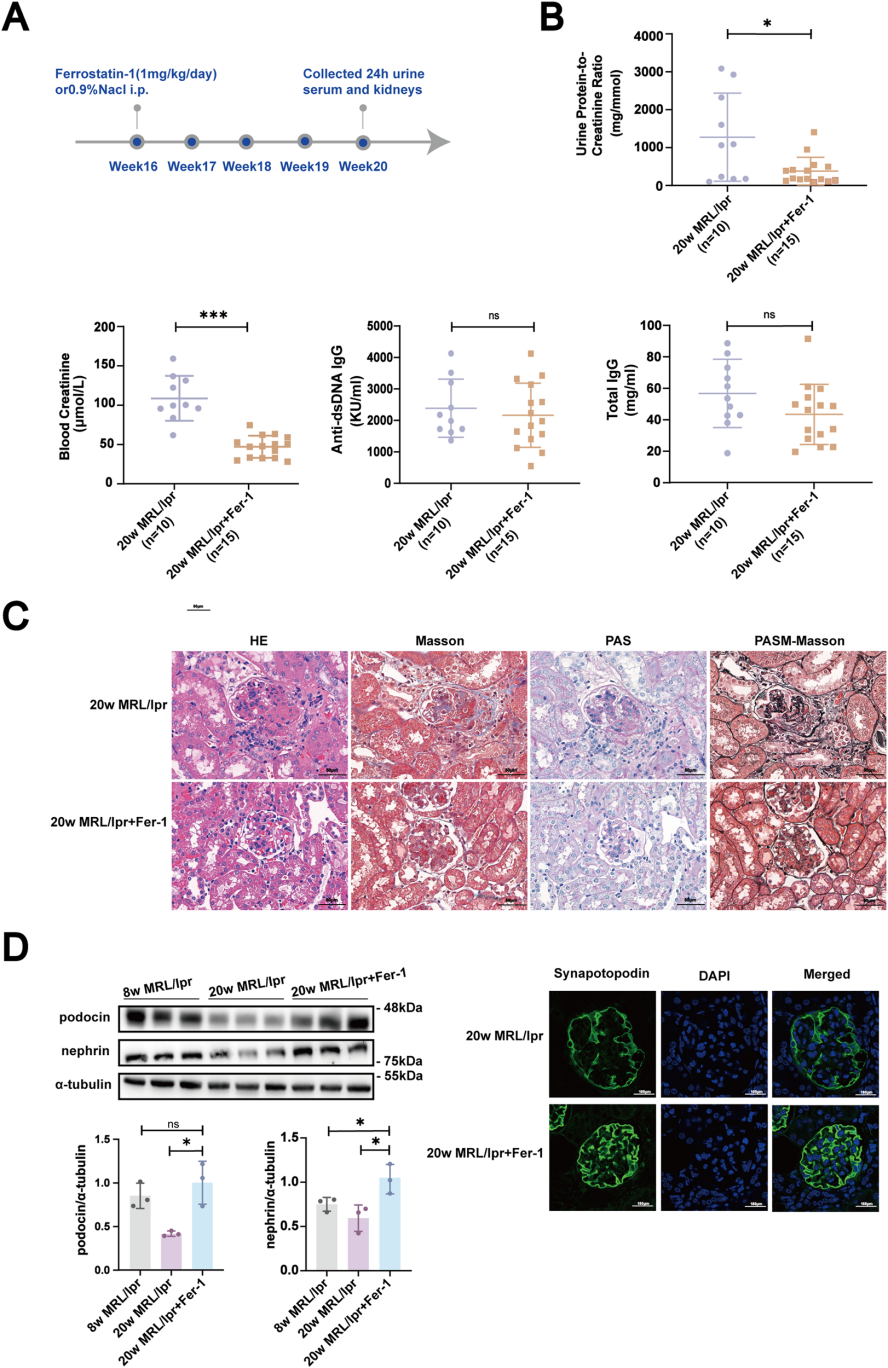
Effect of Ferrostatin-1 on renal damage in MRL/lpr mice with lupus nephritis. **A** Illustration of the experimental timeline for administering Ferrostatin-1 to 20-week-old MRL/lpr mice with established renal injury. **B** Measurement of urinary protein/creatinine ratio and serum creatinine levels post treatment, showing significant reductions, while total IgG and dsDNA levels in blood remained unchanged. **C** Hematoxylin and eosin (HE), Masson’s trichrome, Periodic acid-Schiff (PAS), and PASM (PAS with Methyl Green) staining. Scale bar: 20 μm. **D** Western blot and immunofluorescence analysis demonstrated increased expression of podocyte markers following Ferrostatin-1 treatment, indicating reduced podocyte injury. Scale bar: 20 μm. Data are represented as mean ± SD. *p < 0.05, **p < 0.01, ***p < 0.001.

We administered Ferrostatin-1 treatment to 20-week-old MRL/lpr mice, which previous studies had shown to already exhibit significant renal involvement, including severe lupus nephritis pathology [16, 17]. The experimental schedule, including dosing and treatment duration, was illustrated in Fig. 4A.

Following the administration of Ferrostatin-1, we observed several notable improvements in the treated mice. One of the most significant findings was a marked reduction in the urinary protein/creatinine ratio (Fig. 4B), indicating decreased proteinuria, a common marker of renal dysfunction in lupus nephritis. Additionally, serum creatinine levels, a critical indicator of renal function, were significantly reduced in the Ferrostatin-1 treated group compared to the untreated controls (Fig. 4B). This reduction in serum creatinine suggested an overall improvement in kidney function.

While there were improvements in renal function markers, the levels of total IgG and dsDNA in the blood remained unchanged (Fig. 4B). This observation indicated that Ferrostatin-1’s protective effects did not significantly alter the systemic autoimmune response in these mice.

To determine whether the urinary phenotype reflected pathological findings, we examined the renal pathology of mice. Our observations indicated that Ferrostatin-1 exerted a significant ameliorative effect on the renal histopathological changes in lupus mice with renal injury (Fig. 4C). Specifically, Ferrostatin-1 treatment significantly alleviated glomerular endothelial swelling and markedly decreased the proliferation and expansion of mesangial cells, which are key contributors to the pathogenesis of glomerulosclerosis in LN. Additionally, Ferrostatin-1 treatment was associated with a reduction in vacuole degeneration within renal tubular epithelial cells, indicative of reduced cellular stress and injury. The presence of vacuoles can disrupt normal cellular functions and contribute to the loss of renal function. Furthermore, our histological examination revealed a significant reduction in leukocyte infiltration in the renal interstitium and glomeruli following Ferrostatin-1 administration. Leukocyte infiltration is a common inflammatory response observed in nephropathies and is associated with tissue damage and fibrosis. These findings collectively demonstrate the potential of Ferrostatin-1 in modulating the renal pathological processes associated with nephropathy, highlighting its therapeutic potential in the treatment of LN.

Western blot and immunofluorescence results indicated a significant increase in the expression of podocyte markers in mice after Ferrostatin-1 treatment, suggesting that Ferrostatin-1 effectively alleviated podocyte injury caused by lupus nephritis (Fig. 4D).

### Ferrostatin-1 effectively modulates pathogenic pathways in lupus nephritis

**Fig. 5:**
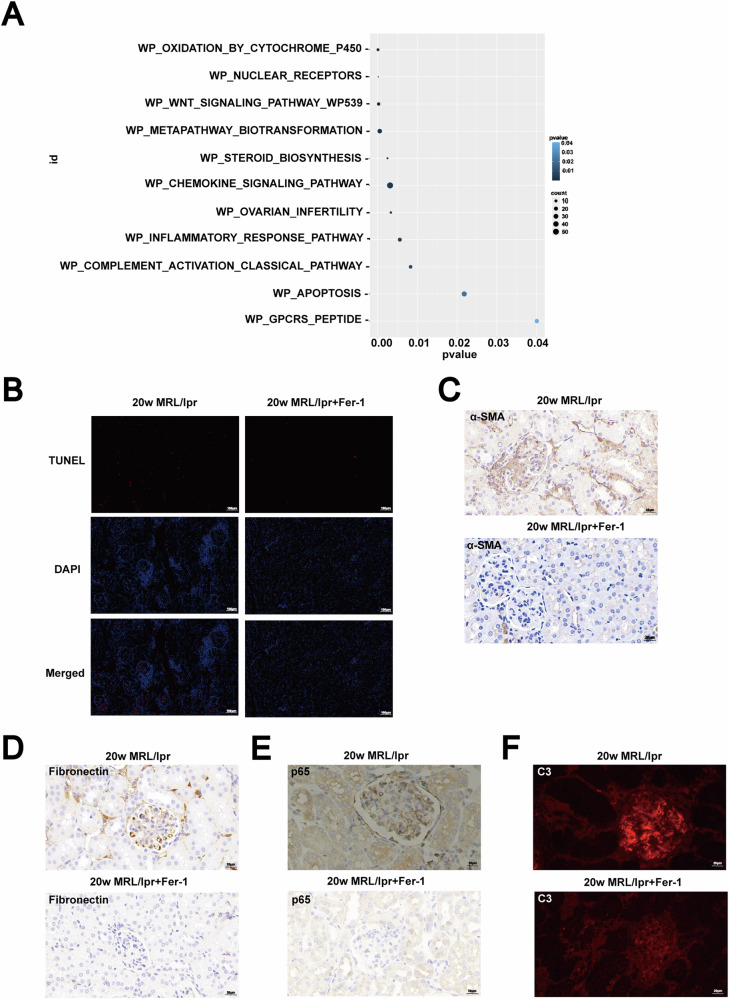
Effect of Ferrostatin-1 on renal damage in MRL/lpr mice with lupus nephritis. **A** Pathway analysis showed downregulation of inflammation, fibrosis, apoptosis, and complement pathways after Ferrostatin-1 application. **B** The TUNEL assay showed that treatment with Ferrostatin-1 significantly reduced renal cell apoptosis in 20-week-old MRL/lpr mice. Scale bar: 100 μm. **C**–**F** Immunohistochemical and western blot analyses revealed that Ferrostatin-1 treatment significantly reduced α-SMA and fibronectin expression, while also decreasing p65 expression and C3 deposition. Scale bar: 20 μm.

Pathway analysis revealed that pathways previously implicated in LN, such as inflammation, fibrosis, apoptosis, and complement activation, were significantly downregulated following Ferrostatin-1 application (Fig. 5A). This finding suggests a comprehensive role for Ferrostatin-1 in modulating the key molecular processes underlying LN pathology.

To validate these results experimentally, treatment with Ferrostatin-1 significantly reduced renal cell apoptosis in 20-week-old MRL/lpr mice, as indicated by decreased levels of apoptotic markers (Fig. 5B). This reduction in apoptosis suggests that Ferrostatin-1 helps preserve renal cell viability and function in the context of LN.

Additionally, Ferrostatin-1 treatment resulted in a notable decrease in the expression of α-SMA, a marker of myofibroblast activation and fibrosis (Fig. 5C). This decrease implies that Ferrostatin-1 effectively inhibits the fibrotic response, which is crucial in preventing the progression of renal scarring and preserving kidney function.

We also observed a significant reduction in the expression of fibronectin, an extracellular matrix protein associated with tissue fibrosis and remodeling (Fig. 5D). This finding further supports the anti-fibrotic effects of Ferrostatin-1, highlighting its potential to mitigate the extracellular matrix accumulation in LN.

Moreover, Ferrostatin-1 treatment led to a substantial decrease in the expression of p65, a subunit of the NF-κB complex involved in inflammatory responses (Fig. 5E). This reduction indicates that Ferrostatin-1 effectively inhibits NF-κB-mediated inflammation, thereby reducing the overall inflammatory effect in the kidneys of LN mice.

In addition to its effects on apoptosis, fibrosis, and inflammation, Ferrostatin-1 treatment significantly reduced C3 deposition in renal tissues (Fig. 5F). C3 deposition is a hallmark of immune complex-mediated kidney damage in lupus nephritis. Histological analysis of kidney sections from 20-week-old MRL/lpr mice treated with Ferrostatin-1 showed a marked decrease in C3 deposition compared to untreated controls (Fig. 5F). This reduction in C3 deposition was associated with improved renal histopathology, including reduced glomerular inflammation and decreased immune complex accumulation.

Collectively, these findings demonstrated that Ferrostatin-1 improved renal damage caused by lupus nephritis through multiple mechanisms: reducing cell apoptosis, inhibiting inflammatory responses, alleviating fibrosis, and mitigating C3 deposition. The broad efficacy of Ferrostatin-1 in modulating these pathogenic pathways underscored its therapeutic potential for treating lupus nephritis.

## Discussion

In the present study, we initially investigated the expression of OTUB1 in LN. A significant reduction of OTUB1 in glomeruli was observed in LN patients by transcriptome analysis and was confirmed by immunofluorescence in both kidney biopsy samples from LN patients and kidney tissues from MRL/lpr mice. The immunohistochemical staining results from the Human Protein Atlas indicated that in normal kidney tissue, OTUB1 expression is high in renal tubules and variable in glomeruli [18]. This variability was consistent with our own staining experiments. Overall, a clear trend emerged, showing a higher incidence of reduced OTUB1 expression in LN cases. This phenomenon was more pronounced in mouse models, potentially due to the presence of complex confounding factors in patient kidney tissue samples, including heterogeneous disease stages and varied medication histories, which could significantly impact OTUB1 expression.

Furthermore, the knockout of the OTUB1 gene via CRISPR-Cas9 resulted in significant podocyte damage, underscoring the pivotal role of OTUB1 in podocyte protection. Numerous studies have demonstrated the cellular protective effects of OTUB1 across various diseases. The upregulation of OTUB1 has been identified as neuroprotective against neuroinflammation-induced NF-κB signaling and neuronal apoptosis [19]. In diabetic cardiomyopathy, OTUB1 interacts with YB-1 to stabilize the YB-1 protein, thereby exerting a protective effect [20]. Additionally, OTUB1 reduces K48-linked ubiquitination and the subsequent degradation of c-IAP1, thus inhibiting necroptotic cell death in hepatocytes [21]. OTUB1 also inhibits the polyubiquitination of TRAF6 and the subsequent activation of the ASK1 signaling pathway, playing a critical protective role in non-alcoholic fatty liver disease [22].

After OTUB1 was knocked out, the expression of SLC7A11 decreased, and oxidative stress markers such as MDA increased, while the expression of cysteine and glutathione decreased. These changes were reversed upon the re-expression of OTUB1. Similar to our results, a previous study reported that OTUB1 interacted with CST1 and enhanced the protein stability of GPX4, thereby reducing the ubiquitination of GPX4, inhibiting ferroptosis, and promoting the metastasis and progression of gastric cancer [23]. The stability of SLC7A11 was maintained by OTUB1, thereby inhibiting ferroptosis in pulmonary artery smooth muscle cells [24]. SLC7A11 protein expression was stabilized through direct interaction with OTUB1, thereby inhibiting ferroptosis. The knockdown of OTUB1 triggered ferroptosis that depended on the expression of SLC7A11 [25, 26].

Moreover, recent studies have demonstrated that OTUB1 acts as a major regulator of SLC7A11 stability in several cancer cell lines, including lung cancer (H1299), neuroblastoma (SK-N-BE(2)C), osteosarcoma (U2OS), and bladder cancer (T24 and UM-UC-3) cells [26]. Our research, along with previous studies, indicated that OTUB1 might play a key role in regulating ferroptosis, with the modulation of SLC7A11 by OTUB1 likely being one of the mechanisms by which it inhibits ferroptosis.

**Fig. 6:**
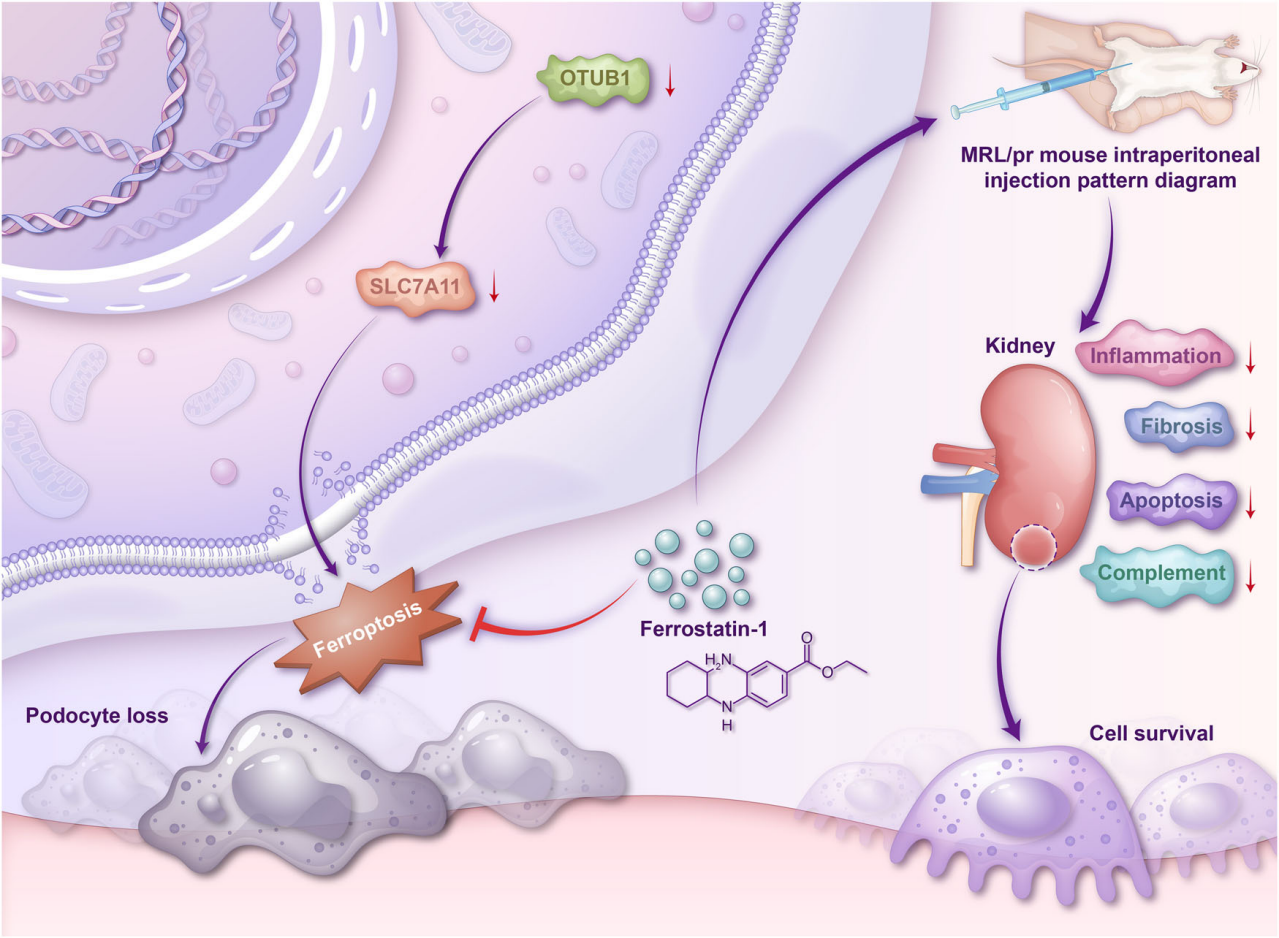
The Protective role of ferrostatin-1 in mitigating OTUB1-dependent ferroptosis in MRL/lpr mice.

Ferroptosis, characterized by iron accumulation and lipid peroxidation, plays a critical role in exacerbating immune cell-mediated pathology in SLE and LN [27–30]. This form of cell death is linked to renal tubular injury through intra-renal dysregulation of iron metabolism [14], highlighting its potential as a target for therapeutic intervention to improve disease outcomes in SLE/LN. In our study, Ferrostatin-1, as a ferroptosis inhibitor, was observed to effectively ameliorate podocyte damage caused by OTUB1 knockout, such as restoring SLC7A11 expression, reducing MDA levels, and increasing cysteine and glutathione levels. Additionally, treatment with Ferrostatin-1 significantly improved renal pathological changes in 20-week-old MRL/lpr mice, including reducing glomerular cell proliferation, matrix expansion, endothelial cell proliferation, inflammatory cell infiltration, and tubular damage. In the present study, we found that Ferrostatin-1 might be linked to its inhibition of inflammatory responses, fibrosis, and the alleviation of cellular apoptosis. Ferrostatin-1 was shown to regulate the TLR4/Trif/type I IFN signaling pathway in heart transplants, reducing cardiomyocyte death and blocking neutrophil recruitment, thereby attenuating inflammation following cardiac transplantation [31]. Ferroptosis-induced liver fibrosis, caused by a high-iron diet or carbon tetrachloride (CCl4) injections, was significantly alleviated by the use of the ferroptosis inhibitor ferrostatin-1 [32]. By suppressing these biological pathways closely associated with lupus nephritis, Ferrostatin-1 not only improved the direct damage to renal tissues but also potentially mitigated further damage caused by the deposition of immune complexes and local inflammatory responses. Figure 6 illustrated the proposed mechanism by which Ferrostatin-1 mitigates OTUB1-dependent ferroptosis and its downstream pathological effects in MRL/lpr mice.

Ferrostatin-1 demonstrated protective effects on podocytes in various pathological models, including acute kidney injury [33], adriamycin-induced focal segmental glomerulosclerosis [34], and diabetic nephropathy [35]. However, the specific mechanisms by which Ferrostatin-1 interacted with OTUB1 and SLC7A11 to modulate podocyte function have not been elucidated to date. Delineating the precise molecular pathways underlying these interactions would represent a direction for future research. While Ferrostatin-1 showed potential as a ferroptosis inhibitor in various models, its clinical applicability may be constrained by concerns such as off-target effects, potential toxicity, and a relatively short half-life. Determining a well-defined therapeutic window to balance efficacy and safety will require further detailed investigations. Exploring these limitations is essential for advancing the translational potential of Ferrostatin-1 as a therapeutic agent.

In our study, we demonstrated that OTUB1 exerts a significant protective effect against lupus nephritis through the targeted regulation of the ferroptosis pathway. Utilizing Ferrostatin-1, a ferroptosis inhibitor, we have shown that it mitigates podocyte injury induced by OTUB1 deficiency, thereby suggesting a promising therapeutic strategy for lupus nephritis.

## Supplementary information

Western blot data

## Data Availability

The data that support the findings of this study are available from the corresponding author upon reasonable request.
