# Supplementary material for: OTUB1 regulation of ferroptosis and the protective role of ferrostatin-1 in lupus nephritis
Source: Cell Death Dis. 2024 Nov 5;15(11):791. doi: 10.1038/s41419-024-07185-5 (PMC11538433; doi:10.1038/s41419-024-07185-5)

1.Figure2A

OTUB1


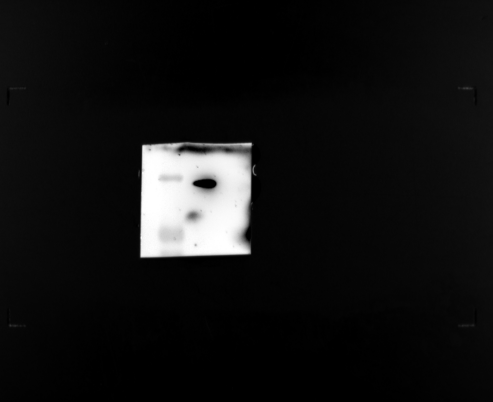

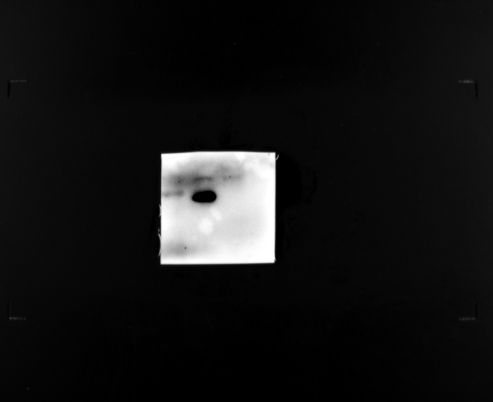

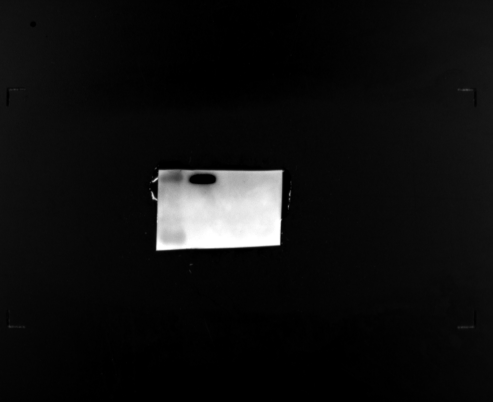


α-tubulin


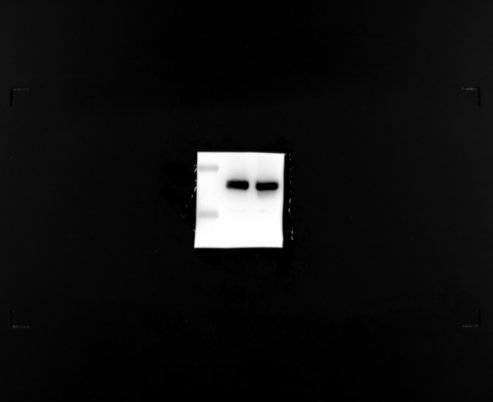

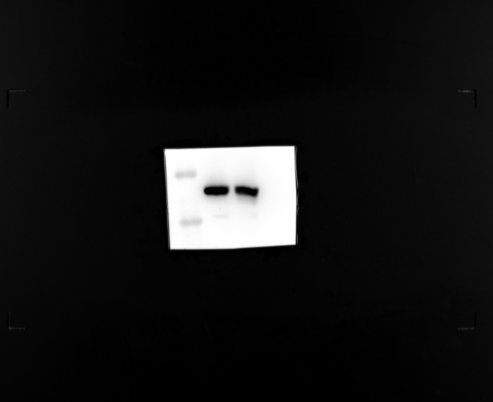

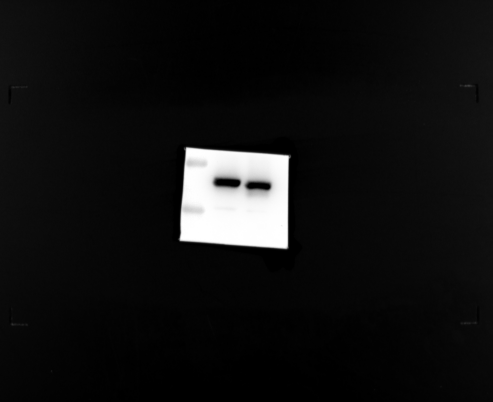


2.Figure2B

OTUB1









podocin


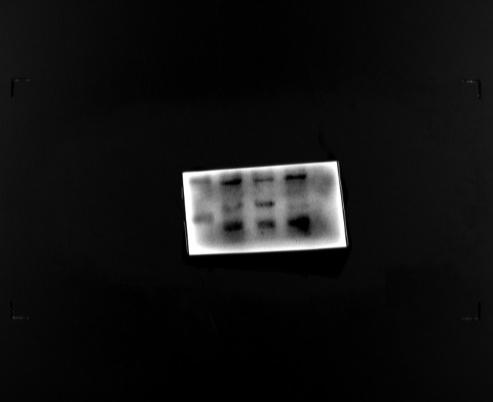






nephrin









α-tubulin









3.Figure2C

OTUB1


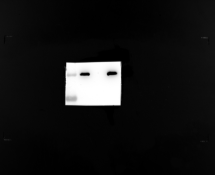

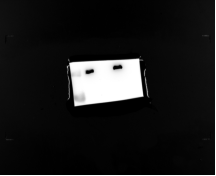

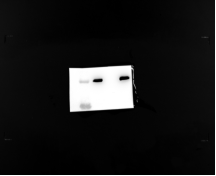


podocin


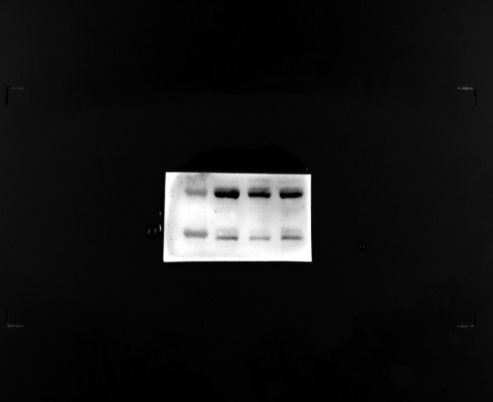

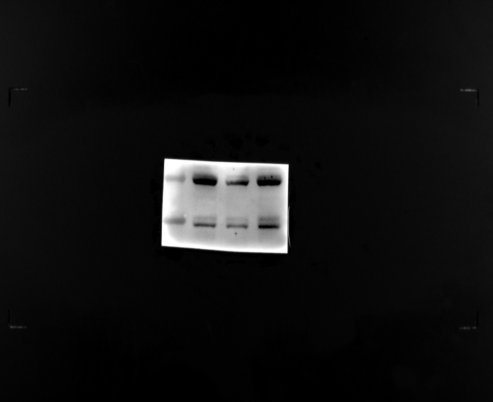

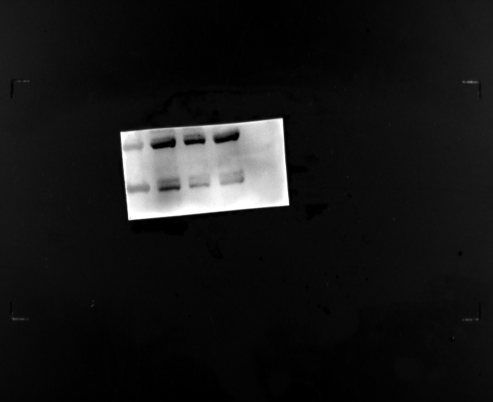


nephrin


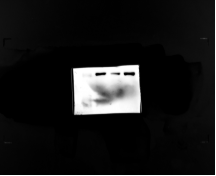

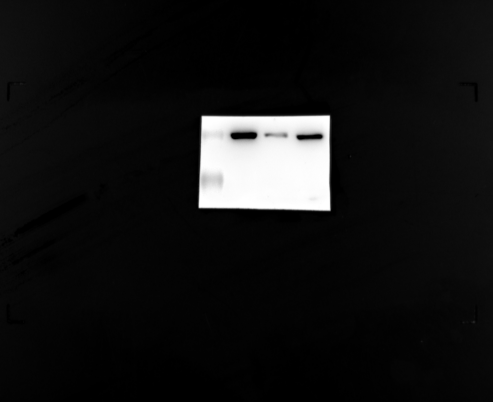

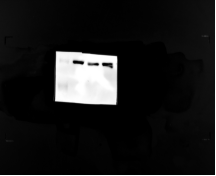


α-tubulin


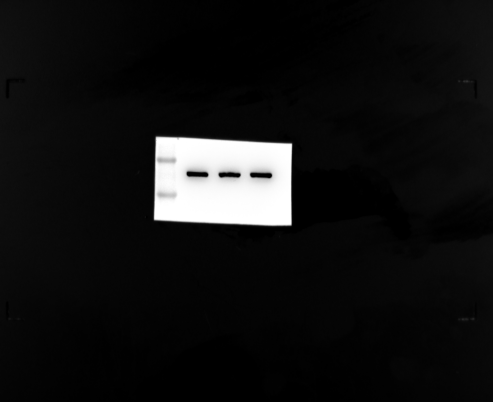

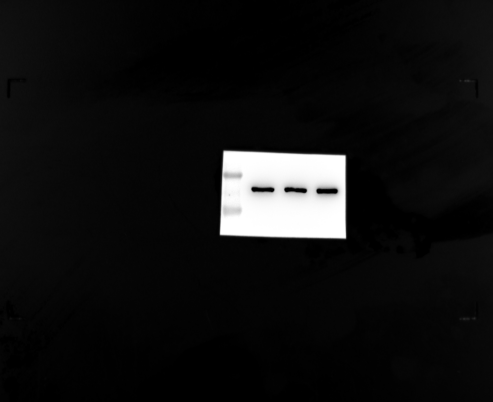

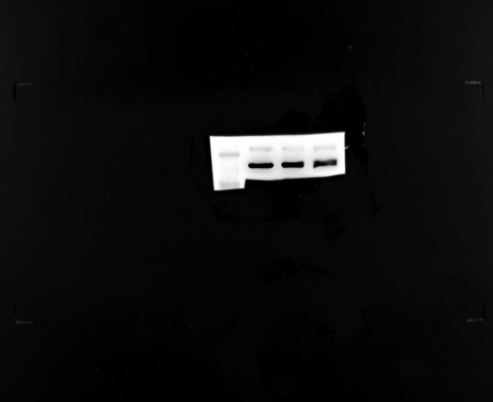


4.Figure2D

OTUB1


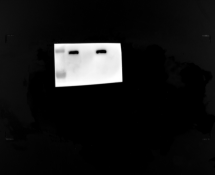

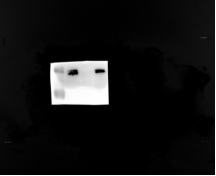

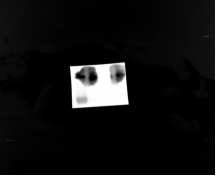


SLC7A11


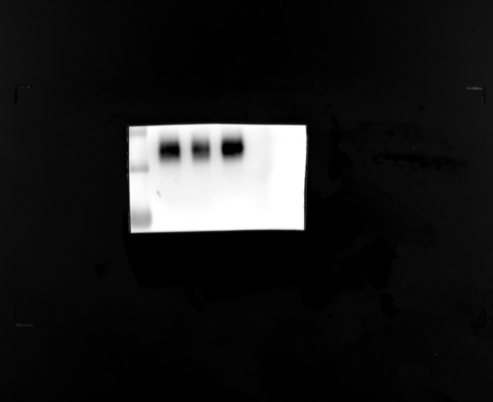

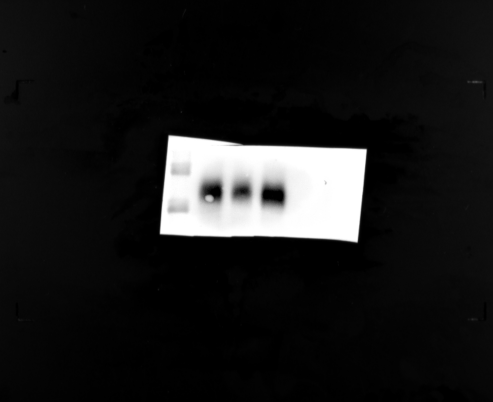

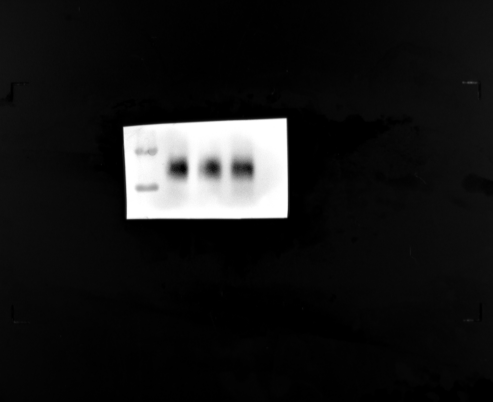


α-tubulin






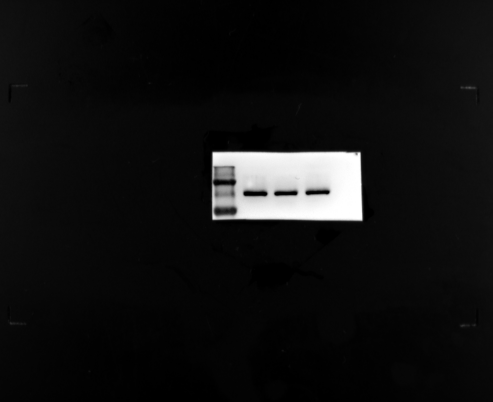


5.Figure3A

SLC7A11


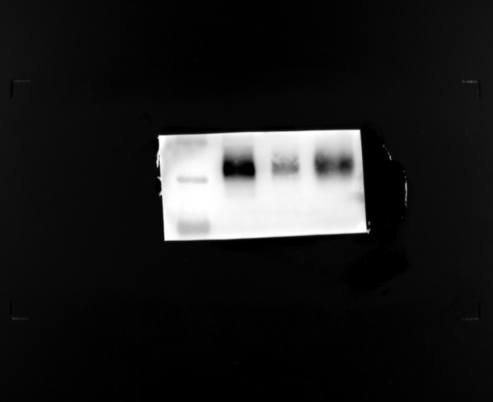

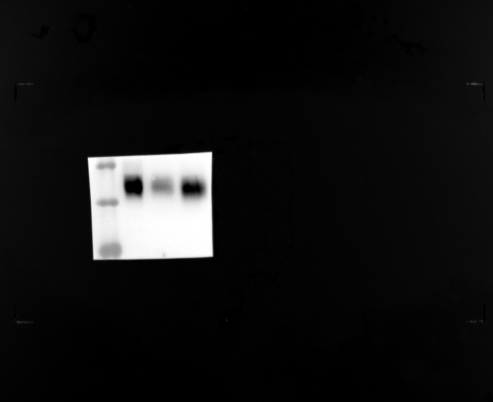

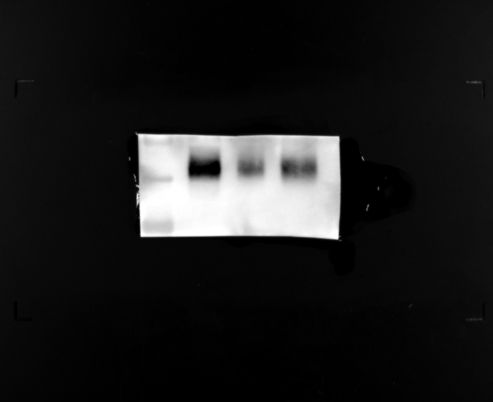


α-tubulin


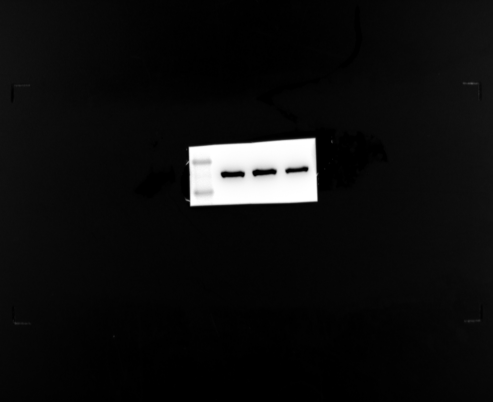

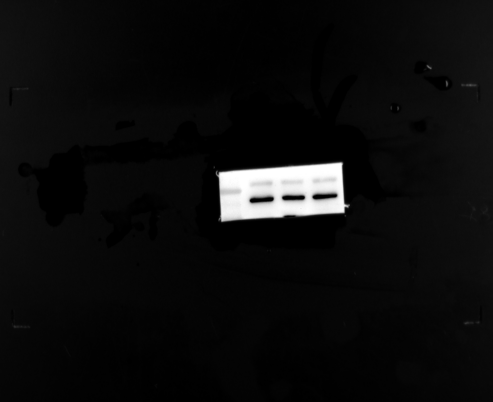

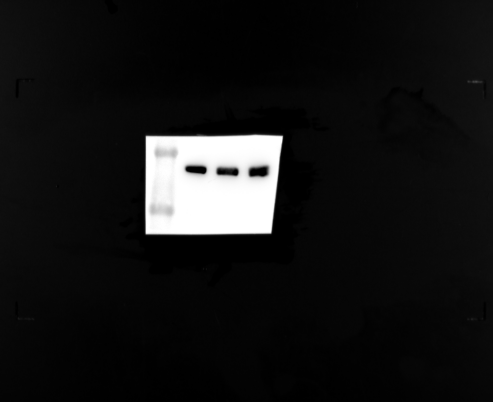


6.Figure3C

OTUB1







podocin


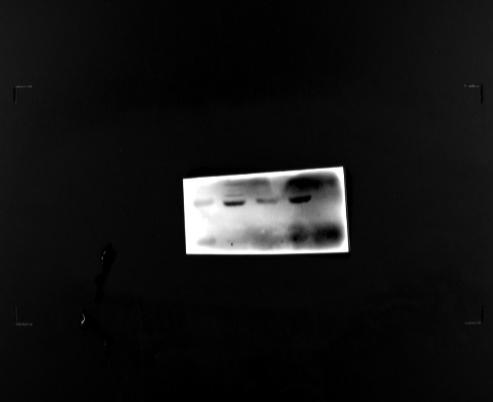



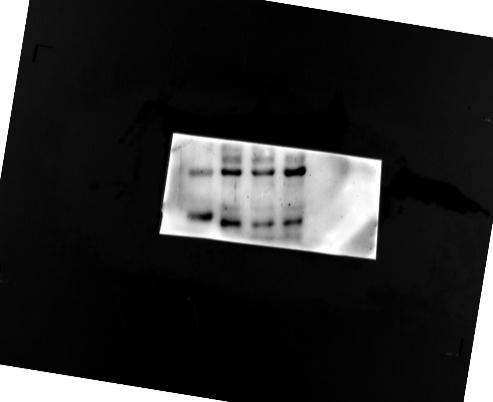



nephrin







α-tubulin









7.Figure4D

podocin


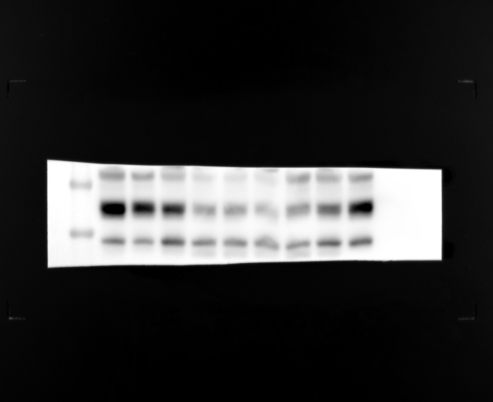

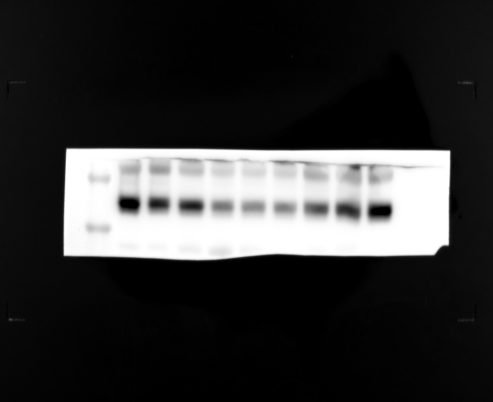

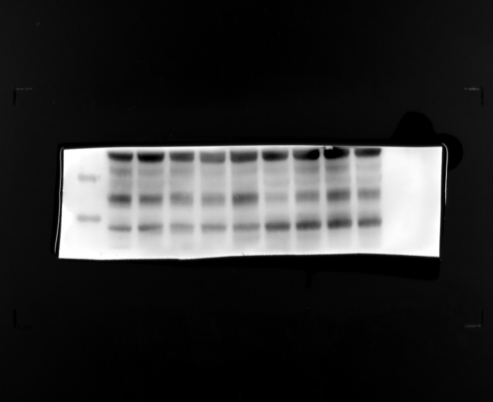


nephrin


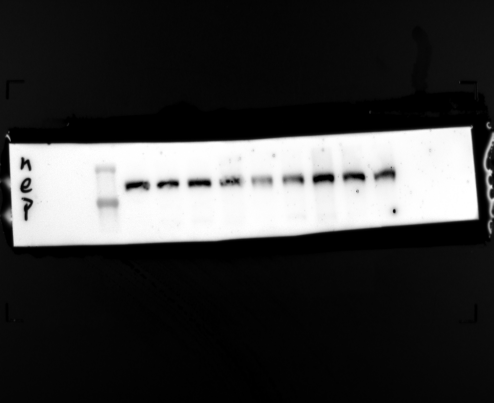

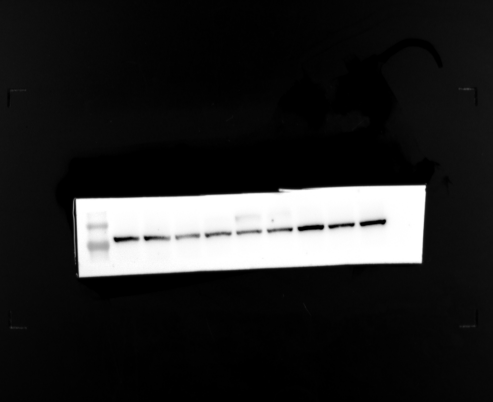

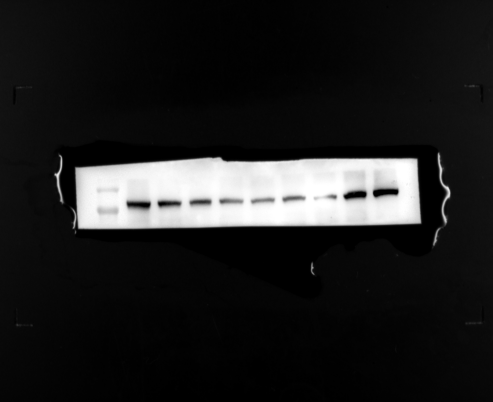


α-tubulin


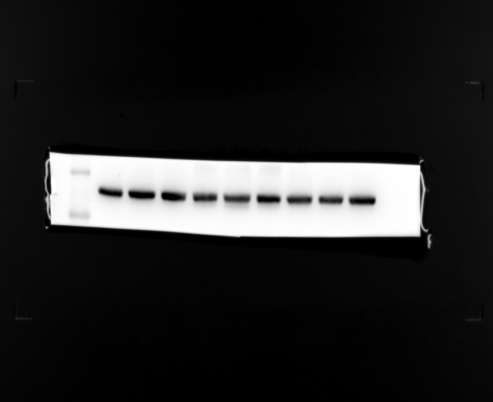

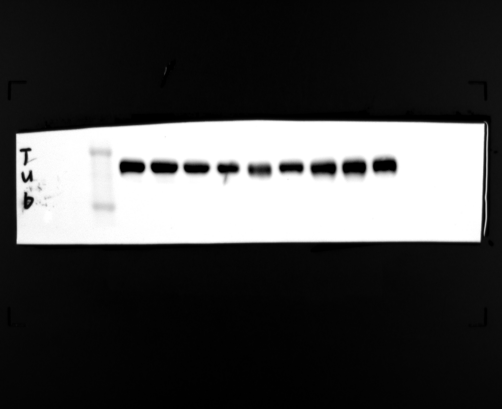

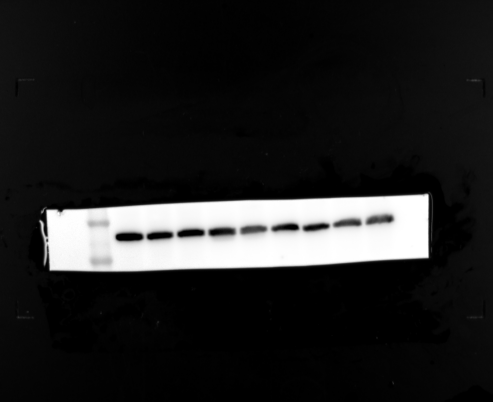

Supplement: Supplementary file 1 — Western blot data [file 41419_2024_7185_MOESM1_ESM.docx]
